# The adequacy of single-incisional thoracoscopic surgery as a first-line endoscopic approach for the management of recurrent primary spontaneous pneumothorax: a retrospective study

**DOI:** 10.1186/1749-8090-7-99

**Published:** 2012-09-29

**Authors:** Chih-Hao Chen, Shih-Yi Lee, Ho Chang, Hung-Chang Liu, Tzu-Ti Hung, Chao-Hung Chen

**Affiliations:** 1 Graduate Institute of Mechanical and Electrical Engineering, National Taipei University of Technology, Taipei City, Taiwan; 2 Department of Thoracic Surgery, Mackay Memorial Hospital, Taipei City, Taiwan; 3 Division of Pulmonary and Critical Care Medicine, Mackay Memorial Hospital, Taipei City, Taiwan; 4 Mackay Medicine, Nursing and Management College, Taipei City, Taiwan

**Keywords:** Pneumothorax, VATS

## Abstract

**Background:**

Thoracoscopic surgery is a commonly used endoscopic surgical treatment approach in patients with primary spontaneous pneumothorax. The conventional thoracoscopic approach utilizes three or more small wounds for surgery. Currently, a single port approach is a potential alternative procedure in general thoracoscopic surgery. We investigated whether a single-port approach is suitable as a first-line endoscopic approach for all patients with primary spontaneous pneumothorax requiring surgery.

**Methods:**

From July 1st, 2008 to Dec 31, 2009, a total of 62 patients was included in this study. All the patients were admitted to our ward because they had surgical indications for surgery. Twenty-six patients underwent conventional three-port thoracoscopic surgery and thirty-six underwent single-port thoracoscopic surgery. All of the clinical data were analyzed retrospectively. Variables were compared and analyzed to determine the outcomes of the different surgical approaches.

**Results:**

The mean age of the 62 patients was 27.2 years. Forty-nine patients were men and thirteen patients were women. The mean time required for the operation was 61.6 minutes. There was one patient who had a recurrence in single-port group and 2 patients had a recurrence in three-port group during the period of follow-up. The average pain scores at 24 and 48 hrs after the operation were similar, but the pain scores at 72 hrs in the single-port group were better than the three-port group. There was no case that required conversion from a single-port to multiple wound approach in this study. There was no immediate postoperative recurrence. The follow-up duration was greater than 12 months.

**Conclusion:**

This study showed that single-port thoracoscopic surgery is a feasible and reasonable first-line endoscopic approach in the surgical treatment of primary spontaneous pneumothorax.

## Background

Thoracoscopic surgery has long been employed as a treatment option for primary spontaneous pneumothorax. Conventional thoracoscopic surgery usually requires multiple small wounds for the purposes of dissection, resection, grasping and the scope itself. In the management of spontaneous pneumothorax, thoracic surgeons commonly utilize three port wounds to complete the procedure. One port wound is for the thorascope itself, another small wound is for any endoscopic grasping instrument that is needed to search for and hold the abnormal lung to be resected and a third small wound is for a stapling, clipping or electrical cauterization. Currently, thoracoscopic surgery can be accomplished through single incision in a variety of common and uncomplicated diseases in the chest [1-5]. However, this technique has not been routinely as a first-line endoscopic approach in most institutions. The purpose of this retrospective study is to evaluate the adequacy of single-incisional thoracoscopic surgery as a first-line endoscopic approach for the surgical treatment of primary spontaneous pneumothorax.

## Methods

From July 1st, 2008 to Dec 31, 2009, a total of 62 patients was included in this study. All of them had the diagnosis of primary spontaneous pneumothorax. There were 49 men and 13 women, and the study period was 18 months. All of the operations were performed by a single thoracic surgeon. In the initial 8 months, conventional three-port thoracoscopic techniques were used in 26 patients. In the following 10 months, a single-port approach was used for the remaining 36 patients. The change at that time was due to modifications in the surgical techniques employed. Therefore, the study population was not assigned randomly. After the initial successful application of this procedure, it was used in consecutive 36 patients.

**Figure 1:**
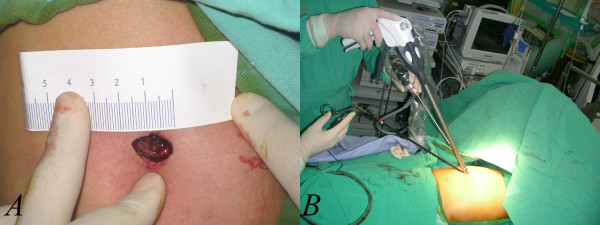
**The single wound approach in the patient with primary spontaneous pneumothorax.** The wound is usually 15 mm in length (**1A**). All of the working tools, including the grasp, stapler and endoscope, were placed through the wound (**1B**). This is the smallest wound achieved with the currently popular endoscopic instruments.

The design of the three-port approach had one scope wound measuring around 12 to 15 mm, usually in the 6th to 7th intercostal space. There was another small 5-mm wound used for any grasping instruments needed and another one of 12 to 15 mm for the placement of a linear stapler, one inch beneath the scapular tip. The instruments we used included a 10.5-mm rigid endoscope with a 0-degree viewing angle, an endoscopic instrument for confirming and sampling the abnormal lung tissue and a linear stapler from another wound for resection. Mechanical (abrasion) pleurodesis was accomplished using a small cleansing pad. After the procedure of wedge resection and pleurodesis, we usually placed a chest tube from Fr. 24 to Fr.28 and then removed it when there were no air leaks and the drainage had become less bloody. The single-port approach design included a single port wound of approximately 15 mm in length, usually in the 5th or 6th intercostal space between the mid-axillary and anterior axillay line. (Figure 1A) A rigid 5-mm endoscope with a 30-degree viewing angle was used. A rigid endoscope, an endoscopic grasping instrument and a linear endoscopic stapler were placed into the pleural cavity through the same wound (Figure 1B). One key goal here is to make the use of a conventional trocar unnecessary, because the rigid nature and cylindrical lumen of such a trocar normally impedes the manipulation of the other instruments. An adequate procedure using multiple straight instruments can not be performed with this sort of trocar in place. Therefore, we simply avoided the use of a trocar in the procedure. Due to the benign nature of the disease, we did not use any trocar or wound protector in the single-port procedure, because cancer seeding is not likely to occur in such a condition. After confirming the location of the abnormal lung tissues, we performed wedge resection and abrasion pleurodesis through the same wound. Chest tubes were removed when there were no air leaks and the tube drainage was no longer bloody.

The visual analogue pain score was recorded 24 hours, 48 hours and 72 hours after the operation. All patients were followed in the outpatient department for at least one year. The indications, side of the pneumothorax, hospital stay, operative time, recurrence and clinical variables were recorded for comparison.

We used SPSS 13.0 for comparison of the clinical variables in the two groups. The Chi-square test and Student *t*-test were used to compare the means of the category and continuous variables. A *p*-value of less than 0.05 was considered significant.

## Results

There were 62 patients included in this study, including 49 men and 13 women. The mean age was 27.2 years. The indications for surgery were recurrence in 49 patients, prolonged air leaks in 9 patients and hemothorax in 4 patients. There were 26 consecutive patients in the three-port group and 36 consecutive patients in the single-port group. The average time required for surgery was 61.6 minutes. The mean time of ICU (intensive care unit) stay was 0.5 days and the mean hospital stay was 4.9 days. The results are listed in Table 1. In the 36 consecutive patients in the single-port group, no patient required conversion to multiple wounds or thoracotomy.

**Table 1:** The clinical variables and outcome after single-port and three-port thoracoscopic surgery

		**Single-port**	**Three-port**	***p*value**
Number		36	26	
Gender	Male	27	22	0.359
	Female	9	4	
Age(year)		29.1	24.5	0.144
Indication(s)				
	recurrence	28	21	0.54
	prolonged air leak	5	4	
	hemothorax	3	1	
Side				
	Right side	22	13	0.384
	Left side	14	13	
ICU stay(day)		0.5	0.4	0.096
HS(day)		4.1	6.2	<0.001
Postoperative recurrence		1 (2.8%)	2(7.7%)	0.16
Wound infection		0 (0%)	1 (3.8%)	
OP time(minutes)		59.3	63.5	0.296
VAS for pain	24 hour	4.0	4.4	0.135
	48 hour	3.2	3.6	0.084
	72 hour	2.5	2.9	0.008
Follow-up(months)		16.3	30.5	<0.001

ICU: intensive care unit, HS: hospital stay, OP: operation, VAS: visual analogue scores for pain,

Immediate postoperative recurrence did not occur in any patient. However, postoperative recurrence of pneumothorax occurred in three patients during the course of follow-up, including one patient in the single-port group and two patients in the three-port group. The patient with recurrence in the single-port group was treated with observation alone because the extent of pneumothorax was minimal in the apex and he remained asymptomatic. Among the two patients with postoperative recurrence in the three-port group, one patient was treated with surgery again because of a prolonged air leak and inadequate lung expansion after tube thoracostomy. One was treated with bedside tube thoracostomy with subsequent chemical pleurodesis. The agent for chemical pleurodesis was Minocycline, 400 mg, for 3 consecutive periods. There were no deaths during the time of follow-up. The average visual analogue pain scores were 4.2 at 24-hours, 3.4 at 48-hours and 2.7 at 72-hours.

In the single-port group, there were 27 men and 9 women, while the three-port group, there were 22 men and 4 women. There was no significant difference (*p* < 0.36). The mean age in the single-port group did not differ from that in the three-port (29.1 vs 24.5 years.; *p* value is 0.14) The operative time in the single-port group was slightly shorter than that in three-port group, but did not reach statistical significance (59.3 minutes vs 63.5 minutes, *p* value is 0.30). The ICU stays of the two groups were quite similar, although not significant. (0.56 days vs 0.44 days, *p* < 0.1) The hospital stay in the single-port group was 4.1 days, which is significantly shorter than the 6.2 days in the three-port group. (*p* <0.001) The complication rate in the three-port group was slightly greater than in the single-port group, but did not reach the level of statistical significance (*p* < 0.07). The visual analogue pain scores at 24 hours and 48 hours were similar. However, the pain score at 72 hours in the single-port group was 2.5, significantly lower than the score of 2.9 in the three-port group (*p* < 0.008).

## Discussion

Thoracoscopic surgery has been developed over a period of many years and became a surgical treatment for primary spontaneous pneumothorax. The most popular approaches include the making of three port wounds for wedge resection and pleurodesis. In recent years, thoracoscopic procedures have been carried out with a single small wound. Uni-port, single-port and single-incisional thoracoscopic surgery have all suggested that there are a number of procedures that can be performed using only a single small wound. Currently, from primary spontensous pneumothorax to lung cancer resection, thoracoscopic surgery using single-port approach has been shown to be feasible [2,4-7]. Taking advantage of the various modifications endoscopic techniques, we can now perform nearly all such procedures without the need of either curved instruments or additional port wounds. However, there is no report on the adequacy of single-port thoracoscopic surgery as a first-line thoracoscopic approach. We thus evaluated the role of such technique as a first-line approach in our study.

**Figure 2:**
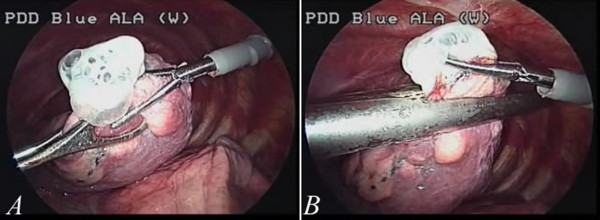
**A case of conventional multi-portal thoracoscopic surgery for pneumothorax.** The lesion site can be approached from different angles (**2A**), which made surgery easier and helped create a three-dimensional working environment. After localization of the abnormal lung, a stapler can be used for resection (**2B**).

**Figure 3:**
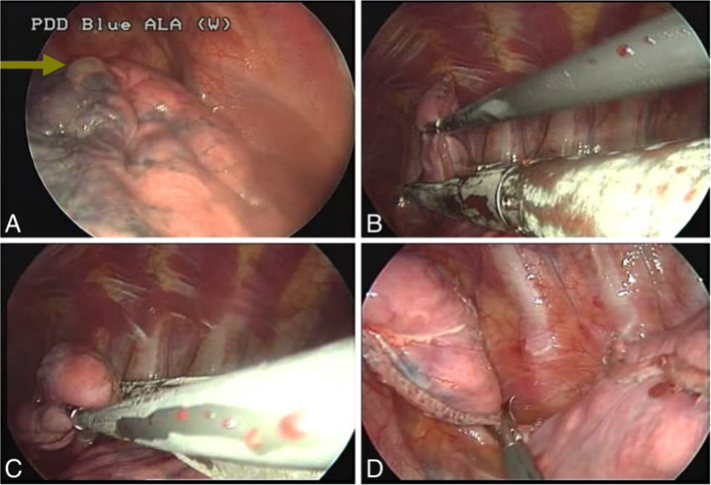
**The setting for single-port thoracoscopic surgery.** We initially confirmed the site of the abnormal lung (the green arrow in **3A**). Then a grasp was used to assist in resection with a linear stapler (**3B**). At times, the instruments may become crossed in the course of completing the procedure, because of the restriction by the tiny wound (**3C**). Scissors may be required to cut the specimen from the remaining lung (**3D**).

**Figure 4:**
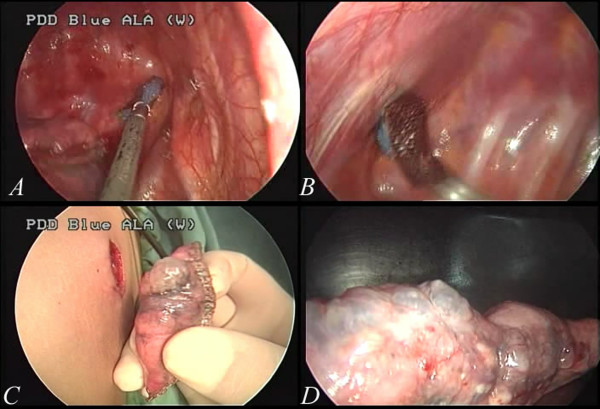
**Pleurodesis with abrasion using a cleansing pad can be performed with instruments positioned at different angles, such as a linear endoscopic grasp to approach the apex (4A), and a long, curved clamp to approach other regions (4B).** In all of the cases, we performed abrasion pleurodesis along with administering minocycline, but we did not perform pleurectomy. The specimen was removed through the small wound (**4C**). Resection with a stapler was shown to be as feasible as in conventional multi-portal thoracoscopic surgery (**4D**).

**Figure 5:**
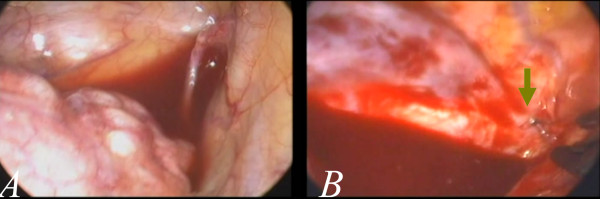
**In some cases recurrent pneumothorax, there may be one or multiple neovascularizations in the apex (5A) that cause excessive bleeding.** With a single-port thoracoscopic surgery, clipping was slightly difficult because the vessel was usually in the most apical region of the pleural space. However, it is still feasible(the green arrow in **5B**).

**Figure 6:**
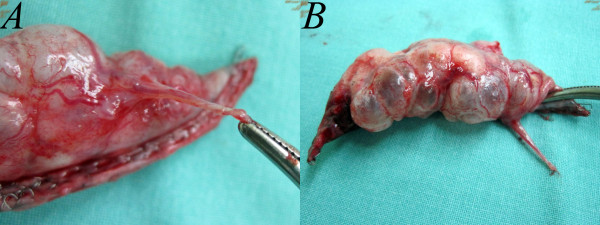
Wedge resection of the apical lung with hyperemic changes (6A) and neovascularization(6B).

In this study, the first 26 patients were treated with conventional three-port thoracoscopic surgery. The port wounds enable the use of a grasping endoscopic instrument by means of an endoscope (usually 10.5 mm, zero degree) and also a linear stapler.(Figure 2A and B) Instruments introduced from different angles are very helpful in the creation of a three-dimensional working environment. Subsequently, we shifted this procedure to single-port approach. Initially we found the procedure difficult to be pferformed with a 15-mm wound. We used a 5-mm endoscope with 30 degrees. As a result, we were able to obtain a better field-of-view from slightly different angles (Figure 1B). However, after confirming the location of the abnormal lung(Figure 3A, green arrow), it was very difficult to keep the endoscopic view stable when a third instrument, usually a linear stapler, placed into the pleural space through the same wound, as in Figure 1B. With repeated adjustments of the endoscopic view and proper arrangement of the instruments, the lesion site (could be) was eventually seen clearly and then a wedge resection could be performed with a stapler (Figure 3B). The instruments have the potential to become crossed due to restriction imposed by a tiny wound (Figure 3C). Thus, sometimes an endoscopic scissors was used to cut the specimen (Figure 3D). After wedge resection, abrasion pleurodesis was performed using a linear, long endoscopic grasping device and a long, curved clamp together with a small cleansing pad (Figure 4A and B). The abnormal lung was completely resected and removed from the tiny wound (Figure 4C and D). During the procedure, we did not utilize any instruments that were not used in the conventional three-port approach. With simple modifications of the technique, thoracoscopic surgery was thus shifted to single-port surgery. In the following consecutive cases, there was no conversion from single-port to multiple port wounds required. Even in those patients with complex or heavy neovascularization (Figure 5A), as well as active bleeding (Figure 5B, the green arrow), the procedures of clipping and hemostasis were performed safely, even with such a tiny wound (Figure 6A and B).

**Figure 7:**
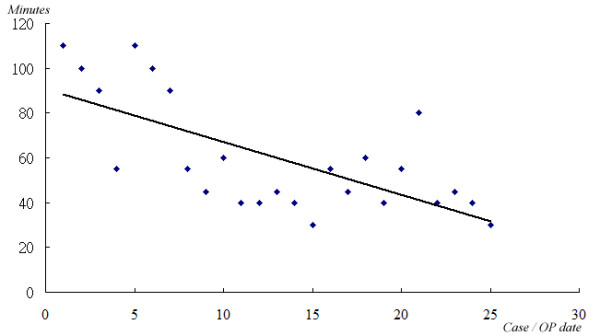
Although the mean operative time required for single-port surgery did not differ from that for conventional multi-port thoracoscopic surgery, it seems obvious that the time required for single-port thoracoscopic surgery decreased substantially along with increasing skill in the procedure.

The operative time required for the single-port approach did not substantially differ from that required for the three-port approach. However, based on our experience, the time period was fairly long when we first began this approach. Later, the speed of the procedure accelerated quickly as it was learned how to position the endoscope and the arrangement of the instruments, in addition to the assistant becoming adequately skilled. In recent uncomplicated cases, the operative time has typically been less than 35 minutes (from skin incision to closure of skin wound). It is clear that the time required for this approach decreased significantly with time (Figure 7).

The pain scores were similar in the first 48 hours after the operation. At 72 hours after the operation, the average pain scores in the single-port group were better than the three-port group (*p* < 0.008) The results may be attributed in part to the smaller number of wounds. No patient required an epidural or intravenous patient-controlled anesthetics. We injected Marcaine for intercostal block in all cases.

It has been anticipated that, in the case of failure, more port wounds would be introduced. In the study period, however, additional wounds were not required. Our confidence developed that the procedure can indeed serve as a first-line approach for thoracoscopic surgery in the management of primary spontaneous pneumothorax. Both experience and technical modifications are mandatory to safely perform these procedures.

One of the two patients with postoperative recurrence in the three-port group was treated by endoscopic surgery again 6 months after the first operation. The apparently greater likelihood of higher recurrence rate in the three-port group than the single-port group may reflect the difference of the follow-up period. The mean follow-up period was 30.5 months in the three-port group and 16.3 months in the single-port group. If the follow-up period similar, the difference between the two procedures may turn out to be smaller.

The minimum effective wound size was 1.5 cm in most of these patients. Due to the pliancy of the lung tissues, the wound can be very small [4,5]. Currently, the wound size depends on the overall size of the three instruments, including a 10-mm stapler, a 5-mm endoscope and a 5-mm grasp, as shown in Figure 1B. If the instruments and the endoscope were smaller, the effective wound size be further decreased [4,8]. The use of multiple rigid trocars, as described by Chen would necessarily result in a larger wound [2].

To the best of our knowledge, this is a first study of single-port thoracoscopic surgery as a first line management of primary spontaneous pneumothorax. This study support the adequacy of the single-port procedure as a first-line endoscopic approach for surgical treatment of primary spontaneous pneumothorax.

## Conclusion

Single-port thoracoscopic approach for primary spontaneous pneumothorax is effective. At the very least, this is a reasonable alternative approach to conventional three-port techniques. Long-term follow-up will be required to confirm the efficacy, safety, cosmetic results and the ultimate patient outcome.

## Consent

Written informed consent was obtained from the patient for publication of this report and any accompanying images.

## Competing interests

The authors declare that they have no competing interests.

## Authors’ contributions

CCH performed the operations and wrote the manuscript. LSY and TTH assisted data collection and analysis. HC, and HCL helped revised the manuscript and provided technical support during operation. CHC provided technical support. All the authors read and approved the final manuscript. Pacific Edit reviewed the manuscript prior to submission.

## References

[B1] Chen CH Huang WC Liu HC Chen TY Surgical outcome of inflammatory pseudotumor in the lung Thorac Cardiovasc Surg 2008 56 214 216 10.1055/s-2007-989340 18481240

[B2] Chen PR Chen CK Lin YS Huang HC Tsai JS Chen CY Single-incision thoracoscopic surgery for primary spontaneous pneumothorax J Cardiothorac Surg 2011 6 58 10.1186/1749-8090-6-58 21507268 PMC3094379

[B3] Prasad R Arthur LG Timmapuri SJ Schwartz MZ Fairbanks TJ Mendelson KG Early experience with single-incision thoracoscopic surgery in the pediatric population J Laparoendosc Adv Surg Tech A 2011 21 189 192 10.1089/lap.2010.0150 21190481

[B4] Chen CH Chang H Tseng PY Hung TT Wu HH A rare case of dysphagia and palpitation caused by the compression exerted by an enormous mediastinal lipoma Rev Port Pneumol 2012 18 149 152 10.1016/j.rppneu.2011.12.003 22261262

[B5] Chen CH Lee SY Chang H Liu HC Huang WC Technical aspects of single-port thoracoscopic surgery for lobectomy J Cardiothorac Surg 2012 7 50 10.1186/1749-8090-7-50 22672719 PMC3431998

[B6] Gonzalez-Rivas D Paradela M Fieira E Velasco C Single-incision video-assisted thoracoscopic lobectomy: Initial results J Thorac Cardiovasc Surg 2011 10.1016/j.jtcvs.2011.07.049 21868042

[B7] Berlanga LA Gigirey O Uniportal video-assisted thoracic surgery for primary spontaneous pneumothorax using a single-incision laparoscopic surgery port: a feasible and safe procedure Surg Endosc 2011 25 2044 2047 10.1007/s00464-010-1470-7 21136111

[B8] Chen CH Chang H Yang LY Liu HC Tsung TT Hung TT A preliminary report of a disposable electrical non-fiberoptic endoscope in thoracoscopic surgery Int J Surg 2012 10 20 24 10.1016/j.ijsu.2011.11.005 22155380

